# Basal metabolic rate as a protective factor against osteoporosis: a multi-cohort longitudinal study from three international aging databases

**DOI:** 10.3389/fnut.2026.1712489

**Published:** 2026-01-22

**Authors:** Yuzhou Cai, Fangyi Dai, Hongyu Li, Yujian Zeng, Peiyu Guo, Tong Zhang

**Affiliations:** 1 Department of Gastrointestinal Surgery, The First Affiliated Hospital of Kunming Medical University, Kunming, China; 2 Haiyuan College, Kunming Medical University, Kunming, Yunnan, China; 3 Department of Orthopedics, The First Affiliated Hospital of Kunming Medical University, Kunming, Yunnan, China

**Keywords:** basal metabolic rate, dose-response, longitudinal cohort, osteoporosis, risk stratification, sarcopenia

## Abstract

**Background:**

Osteoporosis is a major public health burden in aging populations; whether basal metabolic rate (BMR) is independently associated with incident osteoporosis independent of sarcopenia remains unclear.

**Objectives:**

To examine the association between BMR and incident osteoporosis and whether this relationship is modified by sarcopenia status and demographic factors.

**Methods:**

We analyzed 17,836 adults aged ≥45 years from three longitudinal cohorts—ELSA (n = 3,293), HRS (n = 4,498), and SHARE (n = 10,045). BMR was estimated using the Mifflin–St Jeor equation and modeled as quartiles and per 1-SD. Cox proportional hazards models with progressive adjustment were used; restricted cubic splines assessed dose–response. Subgroup/interaction analyses evaluated effect modification. Sensitivity analyses included exclusion of early events, trimming extreme BMR values, complete-case analyses, and cohort- and sex-specific analyses.

**Results:**

Over a median 11.5 years, 1,490 (8.35%) participants developed osteoporosis. Compared with the lowest BMR quartile, the highest quartile had a 37% was associated with lower osteoporosis risk (HR 0.629, 95% CI 0.494–0.800; *p* < 0.001). Each +1 SD increase in BMR was associated with an 18% lower risk (HR 0.823, 95% CI 0.758–0.893; p < 0.001). The association was approximately linear and consistent across cohorts but varied by education (P-interaction = 0.007) and smoking (P-interaction = 0.011). Findings were robust across all sensitivity analyses.

**Conclusion:**

Higher BMR is independently associated with lower incident osteoporosis risk in a linear fashion—beyond sarcopenia and conventional risk factors—with effect modification by education and smoking. As an observational study, causality cannot be established. As an accessible marker of metabolic capacity, BMR may complement existing tools for risk stratification in aging populations.

## Introduction

Osteoporosis has emerged as a critical global health challenge, affecting an estimated 200 million individuals worldwide and accounting for more than 8.9 million fractures annually—approximately one fracture every 3 s (1, 2). Hip and vertebral fractures are associated with excess one-year mortality rates approaching 20% and long-term disability that compromises quality of life and functional independence. As the proportion of adults aged ≥65 years is projected to double between 2010 and 2050, the combined direct and indirect costs of osteoporotic fractures are forecast to exceed US $130 billion per year by the next decade (3). While established risk factors such as advanced age, sex-hormone deficiency, physical inactivity, and inadequate calcium/vitamin D intake remain relevant, contemporary prevention efforts must increasingly address modifiable contributors that transcend these traditional domains.

Emerging evidence suggests that basal metabolic rate (BMR)—the minimum energy expenditure required to maintain vital physiological processes—is associated with skeletal health. Experimental and epidemiological data indicate that BMR closely mirrors systemic metabolic capacity and bone remodeling activity, has been linked to pathways such as AMPK–mTOR–SIRT1/PGC-1α signaling, osteoblast–osteoclast coupling, and muscle–bone crosstalk (4, 5). Clinically, a reduction in muscle mass (sarcopenia) is often paralleled by a decline in BMR, have been associated with bone loss (6, 7). Yet prior studies have largely been cross-sectional, limited to single populations, and unable to disentangle whether low BMR is simply a surrogate for sarcopenia or an independent determinant of osteoporosis risk (8).

To fill this knowledge gap, we leveraged three well-characterized and ethnically diverse longitudinal aging cohorts—the English Longitudinal Study of Ageing (ELSA), the U.S. Health and Retirement Study (HRS), and the Survey of Health, Ageing and Retirement in Europe (SHARE). These datasets collectively offer long follow-up periods, harmonized health measures, and detailed information on body composition, lifestyle factors, and comorbidities. By evaluating incident osteoporosis across nearly two decades of observation, we sought to (i) quantify the prospective association between BMR and fracture-validated osteoporosis, (ii) determine whether this relationship persists after accounting for sarcopenia and other confounders, and (iii) explore potential effect modifiers such as sex, smoking status, and educational attainment. Clarifying the independent contribution of BMR could advance a metabolic framework for osteoporosis prevention, may identify BMR as a potentially measurable marker for early-life and mid-life intervention strategies.

## Materials and methods

### Study design and data sources

This multicenter longitudinal cohort study utilized data from three internationally recognized aging databases: the English Longitudinal Study of Ageing (ELSA, Wave 2–8, 2004–2017, version 9.0, https://www.elsa-project.ac.uk/) (9), the Health and Retirement Study (HRS, Wave 11–14, 2012–2018, version 2022, https://hrs.isr.umich.edu/) (10), and the Survey of Health, Ageing and Retirement in Europe (SHARE, Wave 1–4, 2004–2011, version 8.0.0, https://share-eric.eu/) (11). All contributing studies received ethics approval from their respective review bodies (ELSA: London Multicentre Research Ethics Committee; HRS: University of Michigan Health Sciences and Behavioral Sciences IRB; SHARE: Ethics Council of the Max Planck Society and country-level ethics committees as required).

### Study population and inclusion criteria

Participants aged ≥45 years at baseline were included. Database-specific wave requirements were: HRS (Wave ≥11), ELSA (Wave ≥2), and SHARE (all waves). Exclusion criteria comprised: (1) baseline age <45 years; (2) missing basal metabolic rate (BMR) calculated by Mifflin equation or sarcopenia status at baseline; (3) prevalent osteoporosis at baseline; (4) baseline covariates with missing rates >30% after iterative deletion; and (5) loss to follow-up between baseline and endpoint waves. The baseline waves were defined as Wave 11 for HRS, Wave 2 for ELSA, and Wave 1 for SHARE, with corresponding endpoint waves of 14, 8, and 4, respectively.

### Variable definitions and measurements

BMR was calculated using the Mifflin-St Jeor equation: BMR = [10 × weight (kg)] + [6.25 × height (cm)] − (5 × age (years)] + s, where s = 5 for males and −161 for females. Sarcopenia was defined according to database-specific criteria. Osteoporosis diagnosis was ascertained through self-reported physician diagnosis at each follow-up wave. Covariates included demographic variables (age, gender, education level), lifestyle factors (smoking, drinking status), anthropometric measurements (weight, height, BMI), and comorbidities (hypertension, diabetes mellitus, heart disease, cancer, depression).

### Statistical analysis

Continuous variables were presented as mean ± standard deviation or median (interquartile range), while categorical variables were expressed as frequencies (percentages). Between-group comparisons were performed using Wilcoxon rank-sum test for continuous variables and chi-square test for categorical variables.

BMR was analyzed both as quartiles and as a continuous variable (per 1 SD increase). Kaplan–Meier curves with log-rank tests assessed cumulative osteoporosis incidence across BMR quartiles and sarcopenia status. Cox proportional hazards regression models evaluated the association between BMR and osteoporosis risk, with four progressive adjustment models: Model 1 (crude); Model 2 (adjusted for demographics); Model 3 (additionally adjusted for lifestyle factors); Model 4 (fully adjusted including comorbidities).

Restricted cubic spline (RCS) analysis was employed to explore potential non-linear relationships between BMR and osteoporosis risk. The optimal number of knots (3–5) was determined through a systematic approach: for each model, knots were initially placed at quantile-based positions (3 knots: 25th, 50th, 75th percentiles; 4 knots: 20th, 40th, 60th, 80th percentiles; 5 knots: 10th, 30th, 50th, 70th, 90th percentiles). The final model selection was based on the Akaike Information Criterion (AIC), with the model yielding the lowest AIC value considered optimal. Non-linearity was formally tested using likelihood ratio tests comparing the spline model to a linear model, with *p* < 0.05 indicating significant non-linear associations. The reference point was set at the median BMR value for each analysis. Hazard ratios and 95% confidence intervals were calculated across the BMR range, with adjustment for all covariates in the fully adjusted model.

Subgroup analyses were conducted to identify potential effect modifiers of the BMR-osteoporosis relationship. Participants were stratified by: age (<60 vs. ≥60 years), gender (male vs. female), education level (low, medium, high), smoking status (never/former vs. current), drinking status (no vs. yes), marital status (married vs. others), and sarcopenia status (no vs. yes). For each subgroup, separate Cox proportional hazards models were fitted with BMR analyzed both as quartiles and as a continuous variable (per 1 SD increase). Interaction effects between BMR and each stratifying variable were formally tested using likelihood ratio tests comparing models with and without interaction terms. A *p*-value <0.05 for the interaction term indicated significant effect modification.

Multiple sensitivity analyses validated result robustness: (1) excluding events within 24 months; (2) excluding BMR values beyond 1st and 99th percentiles; (3) complete case analysis; (4) database-specific analyses; (5) varying follow-up truncation points; and (6) gender-stratified analyses.

Missing data were handled using random forest-based multiple imputation (35 imputations). Multicollinearity was assessed using variance inflation factors (VIF) and correlation matrices. All analyses were performed using R version 4.3.0 with significance set at *p* < 0.05 (two-sided).

### Study population and baseline characteristics

**Figure 1:**
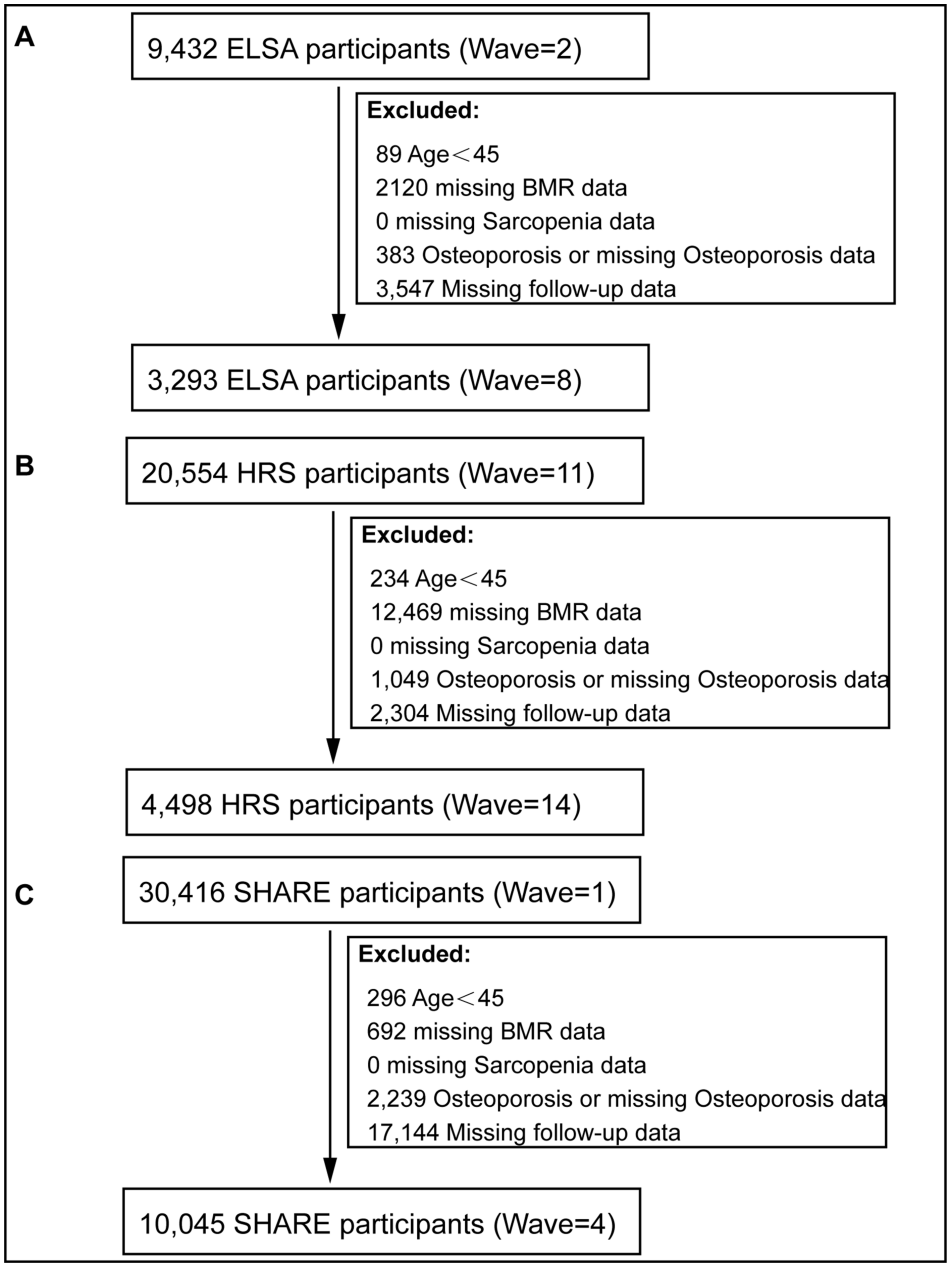
Flowchart of participant selection. **(A)** ELSA cohort selection and exclusions. **(B)** HRS cohort selection and exclusions. **(C)** SHARE cohort selection and exclusions.

A total of 54,385 participants were initially identified from three longitudinal aging cohorts: 9,432 from ELSA (Wave ≥2), 20,554 from HRS (Wave ≥11), and 30,416 from SHARE (Wave ≥1). After applying the inclusion and exclusion criteria, 17,836 participants were included in the final analysis: 3,293 (18.5%) from ELSA, 4,498 (25.2%) from HRS, and 10,045 (56.3%) from SHARE (Figure 1). The primary reasons for exclusion varied across cohorts but consistently included age <45 years, missing BMR or sarcopenia data, prevalent osteoporosis at baseline, and loss to follow-up. Notably, the largest exclusion in each cohort was due to missing follow-up data: 3,547 (37.6%) in ELSA, 2,304 (11.2%) in HRS, and 17,144 (56.4%) in SHARE.

The baseline characteristics stratified by sarcopenia status revealed significant differences across all three cohorts (Tables 1–3). The overall prevalence of sarcopenia was 10.9% in ELSA, 8.4% in HRS, and 8.3% in SHARE. Participants with sarcopenia were consistently older across all cohorts, with median ages of 68 years (IQR: 61–74) in ELSA, 74 years (IQR: 65–80) in HRS, and 77 years (IQR: 68–80) in SHARE, compared to their non-sarcopenic counterparts (all *p* < 0.001). Female predominance was observed in the sarcopenia group across all cohorts: 74.4% in ELSA, 62.4% in HRS, and 60.7% in SHARE. Participants with sarcopenia demonstrated significantly lower BMR values across all databases, with median values of 1,256.25 kcal/day in ELSA, 1,295.19 kcal/day in HRS, and 1,266.50 kcal/day in SHARE, representing substantial reductions compared to non-sarcopenic participants (all *p* < 0.001, SMD ranging from 0.575 to 0.652).

**Table 1:** Baseline characteristics by sarcopenia status in ELSA cohort.

Characteristics	Level	Overall (*n =* 3,293)	No (*n* = 2,933)	Yes (*n* = 360)	*p*-value	SMD
Age (years) [median (Q1, Q3)]		61.00 [57.00, 68.00]	61.00 [57.00, 67.00]	68.00 [61.00, 74.00]	<0.001	0.698
Gender	Female	1815 (55.1)	1,547 (52.7)	268 (74.4)	<0.001	0.463
	Male	1,478 (44.9)	1,386 (47.3)	92 (25.6)		
Education level	Low	1,008 (30.6)	836 (28.5)	172 (47.8)	<0.001	0.459
	Medium	1,698 (51.6)	1,539 (52.5)	159 (44.2)		
	High	587 (17.8)	558 (19.0)	29 (8.1)		
Marital status	Divorced	395 (12.0)	344 (11.7)	51 (14.2)	<0.001	0.349
	Married	2,352 (71.4)	2,143 (73.1)	209 (58.1)		
	Single	148 (4.5)	127 (4.3)	21 (5.8)		
	Widowed	398 (12.1)	319 (10.9)	79 (21.9)		
Current smoking	No	2,896 (87.9)	2,577 (87.9)	319 (88.6)	0.732	0.023
	Yes	397 (12.1)	356 (12.1)	41 (11.4)		
Current drinking	No	224 (6.8)	178 (6.1)	46 (12.8)	<0.001	0.231
	Yes	3,069 (93.2)	2,755 (93.9)	314 (87.2)		
Weight (kg) [median (Q1, Q3)]		77.10 [66.90, 87.00]	77.50 [67.60, 87.50]	72.05 [61.77, 82.25]	<0.001	0.346
Height (m) [median (Q1, Q3)]		1.66 [1.60, 1.73]	1.67 [1.60, 1.74]	1.60 [1.54, 1.65]	<0.001	0.741
BMI (kg/m^2^) [median (Q1, Q3)]		27.46 [24.80, 30.72]	27.42 [24.81, 30.64]	27.85 [24.50, 31.30]	0.493	0.056

Data are presented as median (Q1, Q3) for continuous variables and *n* (%) for categorical variables. *p*-values were calculated using Wilcoxon rank-sum test for continuous variables and chi-square test for categorical variables. SMD: standardized mean difference.

**Table 2:** Baseline characteristics by sarcopenia status in HRS cohort.

Characteristics	Level	Overall (*n =* 4,498)	No (*n* = 4,120)	Yes (*n* = 378)	*p*-value	SMD
Age (years) [median (Q1, Q3)]		63.00 [56.00, 72.00]	62.00 [56.00, 71.00]	74.00 [65.00, 80.00]	<0.001	0.876
Gender	Female	2,493 (55.4)	2,257 (54.8)	236 (62.4)	0.004	0.156
	Male	2005 (44.6)	1863 (45.2)	142 (37.6)		
Education level	Low	726 (16.1)	614 (14.9)	112 (29.6)	<0.001	0.371
	Medium	2,619 (58.2)	2,423 (58.8)	196 (51.9)		
	High	1,153 (25.6)	1,083 (26.3)	70 (18.5)		
Marital status	Divorced	782 (17.4)	725 (17.6)	57 (15.1)	<0.001	0.384
	Married	2,833 (63.0)	2,633 (63.9)	200 (52.9)		
	Single	275 (6.1)	257 (6.2)	18 (4.8)		
	Widowed	608 (13.5)	505 (12.3)	103 (27.2)		
Current smoking	No	3,875 (86.1)	3,532 (85.7)	343 (90.7)	0.006	0.156
	Yes	623 (13.9)	588 (14.3)	35 (9.3)		
Current drinking	No	1754 (39.0)	1,553 (37.7)	201 (53.2)	<0.001	0.315
	Yes	2,744 (61.0)	2,567 (62.3)	177 (46.8)		
Weight (kg) [median (Q1, Q3)]		82.55 [70.67, 95.07]	83.32 [71.49, 95.64]	72.71 [63.48, 86.00]	<0.001	0.486
Height (m) [median (Q1, Q3)]		1.66 [1.59, 1.74]	1.66 [1.60, 1.74]	1.61 [1.54, 1.69]	<0.001	0.506
BMI (kg/m^2^) [median (Q1, Q3)]		28.20 [25.10, 32.10]	28.30 [25.20, 32.20]	27.30 [23.50, 31.15]	<0.001	0.217

Data are presented as median (Q1, Q3) for continuous variables and *n* (%) for categorical variables. *p*-values were calculated using Wilcoxon rank-sum test for continuous variables and chi-square test for categorical variables. SMD: Standardized mean difference.

**Table 3:** Baseline characteristics by sarcopenia status in SHARE cohort.

Characteristics	Level	Overall (*n* = 10,045)	No (*n* = 9,212)	Yes (*n* = 833)	*p*-value	SMD
Age (years) [median (Q1, Q3)]		61.00 [55.00, 69.00]	61.00 [55.00, 67.00]	77.00 [68.00, 80.00]	<0.001	1.327
Gender	Female	5,354 (53.3)	4,848 (52.6)	506 (60.7)	<0.001	0.164
	Male	4,691 (46.7)	4,364 (47.4)	327 (39.3)		
Education level	Low	4,791 (47.7)	4,186 (45.4)	605 (72.6)	<0.001	0.582
	Medium	3,047 (30.3)	2,899 (31.5)	148 (17.8)		
	High	2,207 (22.0)	2,127 (23.1)	80 (9.6)		
Marital status	Divorced	773 (7.7)	726 (7.9)	47 (5.6)	<0.001	0.544
	Married	7,525 (74.9)	7,047 (76.5)	478 (57.4)		
	Single	549 (5.5)	501 (5.4)	48 (5.8)		
	Widowed	1,198 (11.9)	938 (10.2)	260 (31.2)		
Current smoking	No	8,262 (82.2)	7,511 (81.5)	751 (90.2)	<0.001	0.249
	Yes	1783 (17.8)	1701 (18.5)	82 (9.8)		
Current drinking	No	2,174 (21.6)	1847 (20.0)	327 (39.3)	<0.001	0.430
	Yes	7,871 (78.4)	7,365 (80.0)	506 (60.7)		
Weight (kg) [median (Q1, Q3)]		74.00 [65.00, 83.00]	74.00 [65.00, 84.00]	70.00 [63.00, 80.00]	<0.001	0.223
Height (m) [median (Q1, Q3)]		1.68 [1.62, 1.75]	1.68 [1.62, 1.75]	1.64 [1.58, 1.70]	<0.001	0.549

Data are presented as median (Q1, Q3) for continuous variables and *n* (%) for categorical variables. *p*-values were calculated using Wilcoxon rank-sum test for continuous variables and chi-square test for categorical variables. SMD: Standardized mean difference.

When stratified by BMR quartiles, distinct patterns emerged across the cohorts (Tables 4–6). The BMR distributions showed clear gender disparities, with females predominantly represented in the lowest quartile (Q1: 99.3% in ELSA, 97.8% in HRS, 98.6% in SHARE) and males in the highest quartile (Q4: 93.3% in ELSA, 86.6% in HRS, 94.5% in SHARE). An inverse relationship was observed between age and BMR quartiles, with participants in Q1 being older (median ages: 65, 69, and 65 years in ELSA, HRS, and SHARE, respectively) compared to those in Q4 (median ages: 59, 59, and 58 years, respectively; all *p* < 0.001). As expected, anthropometric measurements including weight, height, and BMI increased progressively from Q1 to Q4 across all cohorts. The prevalence of comorbidities showed mixed patterns: diabetes mellitus and hypertension generally increased with higher BMR quartiles, while depression showed an inverse relationship, being more prevalent in lower BMR quartiles across all three databases.

**Table 4:** Baseline characteristics by BMR quartiles in ELSA cohort.

Characteristics	Level	Overall (*n* = 3,293)	Q1 (Lowest) (*n* = 824)	Q2 (*n* = 823)	Q3 (*n* = 823)	Q4 (Highest) (*n* = 823)	*p*-value	SMD
Age (years) [median (Q1, Q3)]		61.00 [57.00, 68.00]	65.00 [58.00, 71.00]	61.00 [57.00, 68.00]	62.00 [57.00, 68.00]	59.00 [55.00, 64.00]	<0.001	0.357
Gender	Female	1815 (55.1)	818 (99.3)	689 (83.7)	253 (30.7)	55 (6.7)	<0.001	1.995
	Male	1,478 (44.9)	6 (0.7)	134 (16.3)	570 (69.3)	768 (93.3)		
Education level	Low	1,008 (30.6)	324 (39.3)	288 (35.0)	217 (26.4)	179 (21.7)	<0.001	0.254
	Medium	1,698 (51.6)	395 (47.9)	417 (50.7)	444 (53.9)	442 (53.7)		
	High	587 (17.8)	105 (12.7)	118 (14.3)	162 (19.7)	202 (24.5)		
Marital status	Divorced	395 (12.0)	123 (14.9)	105 (12.8)	83 (10.1)	84 (10.2)	<0.001	0.334
	Married	2,352 (71.4)	487 (59.1)	565 (68.7)	634 (77.0)	666 (80.9)		
	Single	148 (4.5)	28 (3.4)	52 (6.3)	36 (4.4)	32 (3.9)		
	Widowed	398 (12.1)	186 (22.6)	101 (12.3)	70 (8.5)	41 (5.0)		
Current smoking	No	2,896 (87.9)	715 (86.8)	718 (87.2)	727 (88.3)	736 (89.4)	0.347	0.047
	Yes	397 (12.1)	109 (13.2)	105 (12.8)	96 (11.7)	87 (10.6)		
Current drinking	No	224 (6.8)	71 (8.6)	56 (6.8)	57 (6.9)	40 (4.9)	0.025	0.076
	Yes	3,069 (93.2)	753 (91.4)	767 (93.2)	766 (93.1)	783 (95.1)		
Weight (kg) [median (Q1, Q3)]		77.10 [66.90, 87.00]	61.70 [57.10, 66.10]	74.00 [68.90, 79.40]	79.90 [74.60, 86.60]	92.30 [86.50, 101.80]	<0.001	1.969
Height (m) [median (Q1, Q3)]		1.66 [1.60, 1.73]	1.57 [1.53, 1.61]	1.63 [1.59, 1.66]	1.69 [1.65, 1.73]	1.77 [1.73, 1.81]	<0.001	1.804

BMR quartiles were defined based on database-specific distributions. Q1 represents the lowest quartile and Q4 the highest. Data are presented as median (Q1, Q3) for continuous variables and *n* (%) for categorical variables. *p*-values were calculated using Wilcoxon rank-sum test for continuous variables and chi-square test for categorical variables. SMD: standardized mean difference.

**Table 5:** Baseline characteristics by BMR quartiles in HRS cohort.

Characteristics	Level	Overall (*n* = 4,498)	Q1 (Lowest) (*n* = 1,125)	Q2 (*n* = 1,124)	Q3 (*n* = 1,124)	Q4 (Highest) (*n* = 1,125)	*p*-value	SMD
Age (years) [median (Q1, Q3)]		63.00 [56.00, 72.00]	69.00 [59.00, 76.00]	63.00 [57.00, 72.00]	62.00 [56.00, 72.00]	59.00 [55.00, 67.00]	<0.001	0.374
Gender	Female	2,493 (55.4)	1,100 (97.8)	848 (75.4)	394 (35.1)	151 (13.4)	<0.001	1.448
	Male	2005 (44.6)	25 (2.2)	276 (24.6)	730 (64.9)	974 (86.6)		
Education level	Low	726 (16.1)	231 (20.5)	200 (17.8)	181 (16.1)	114 (10.1)	<0.001	0.171
	Medium	2,619 (58.2)	651 (57.9)	650 (57.8)	640 (56.9)	678 (60.3)		
	High	1,153 (25.6)	243 (21.6)	274 (24.4)	303 (27.0)	333 (29.6)		
Marital status	Divorced	782 (17.4)	188 (16.7)	199 (17.7)	201 (17.9)	194 (17.2)	<0.001	0.305
	Married	2,833 (63.0)	608 (54.0)	677 (60.2)	743 (66.1)	805 (71.6)		
	Single	275 (6.1)	62 (5.5)	77 (6.9)	74 (6.6)	62 (5.5)		
	Widowed	608 (13.5)	267 (23.7)	171 (15.2)	106 (9.4)	64 (5.7)		
Current smoking	No	3,875 (86.1)	980 (87.1)	965 (85.9)	952 (84.7)	978 (86.9)	0.322	0.040
	Yes	623 (13.9)	145 (12.9)	159 (14.1)	172 (15.3)	147 (13.1)		
Current drinking	No	1754 (39.0)	520 (46.2)	457 (40.7)	432 (38.4)	345 (30.7)	<0.001	0.169
	Yes	2,744 (61.0)	605 (53.8)	667 (59.3)	692 (61.6)	780 (69.3)		
Weight (kg) [median (Q1, Q3)]		82.55 [70.67, 95.07]	64.86 [59.06, 70.13]	79.33 [72.39, 85.33]	86.36 [79.74, 94.28]	103.33 [94.35, 114.03]	<0.001	2.057
Height (m) [median (Q1, Q3)]		1.66 [1.59, 1.74]	1.57 [1.52, 1.62]	1.63 [1.59, 1.68]	1.70 [1.64, 1.75]	1.77 [1.71, 1.82]	<0.001	1.458

Data are presented as median (Q1, Q3) for continuous variables and *n* (%) for categorical variables. *p*-values were calculated using Wilcoxon rank-sum test for continuous variables and chi-square test for categorical variables. SMD: Standardized mean difference.

**Table 6:** Baseline characteristics by BMR quartiles in SHARE cohort.

Characteristics	Level	Overall (*n* = 10,045)	Q1 (Lowest) (*n* = 2,512)	Q2 (*n* = 2,512)	Q3 (*n* = 2,512)	Q4 (Highest) (*n* = 2,509)	*p*-value	SMD
Age (years) [median (Q1, Q3)]		61.00 [55.00, 69.00]	65.00 [57.00, 73.00]	61.00 [55.00, 68.00]	62.00 [56.00, 69.00]	58.00 [54.00, 64.00]	<0.001	0.368
Gender	Female	5,354 (53.3)	2,477 (98.6)	2039 (81.2)	700 (27.9)	138 (5.5)	<0.001	2.027
	Male	4,691 (46.7)	35 (1.4)	473 (18.8)	1812 (72.1)	2,371 (94.5)		
Education level	Low	4,791 (47.7)	1,391 (55.4)	1,296 (51.6)	1,191 (47.4)	913 (36.4)	<0.001	0.208
	Medium	3,047 (30.3)	651 (25.9)	700 (27.9)	776 (30.9)	920 (36.7)		
	High	2,207 (22.0)	470 (18.7)	516 (20.5)	545 (21.7)	676 (26.9)		
Marital status	Divorced	773 (7.7)	196 (7.8)	208 (8.3)	174 (6.9)	195 (7.8)	<0.001	0.339
	Married	7,525 (74.9)	1,572 (62.6)	1858 (74.0)	2018 (80.3)	2077 (82.8)		
	Single	549 (5.5)	154 (6.1)	122 (4.9)	139 (5.5)	134 (5.3)		
	Widowed	1,198 (11.9)	590 (23.5)	324 (12.9)	181 (7.2)	103 (4.1)		
Current smoking	No	8,262 (82.2)	2,168 (86.3)	2,105 (83.8)	2000 (79.6)	1989 (79.3)	<0.001	0.112
	Yes	1783 (17.8)	344 (13.7)	407 (16.2)	512 (20.4)	520 (20.7)		
Current drinking	No	2,174 (21.6)	789 (31.4)	649 (25.8)	471 (18.8)	265 (10.6)	<0.001	0.293
	Yes	7,871 (78.4)	1723 (68.6)	1863 (74.2)	2041 (81.2)	2,244 (89.4)		
Weight (kg) [median (Q1, Q3)]		74.00 [65.00, 83.00]	60.00 [56.00, 65.00]	70.00 [66.00, 76.00]	77.00 [72.00, 82.00]	89.00 [83.00, 96.00]	<0.001	1.957
Height (m) [median (Q1, Q3)]		1.68 [1.62, 1.75]	1.60 [1.56, 1.64]	1.65 [1.61, 1.69]	1.71 [1.67, 1.75]	1.78 [1.74, 1.82]	<0.001	1.702

Data are presented as median (Q1, Q3) for continuous variables and *n* (%) for categorical variables. *p*-values were calculated using Wilcoxon rank-sum test for continuous variables and chi-square test for categorical variables. SMD: Standardized mean difference.

### Distribution of basal metabolic rate by sarcopenia status

**Figure 2:**
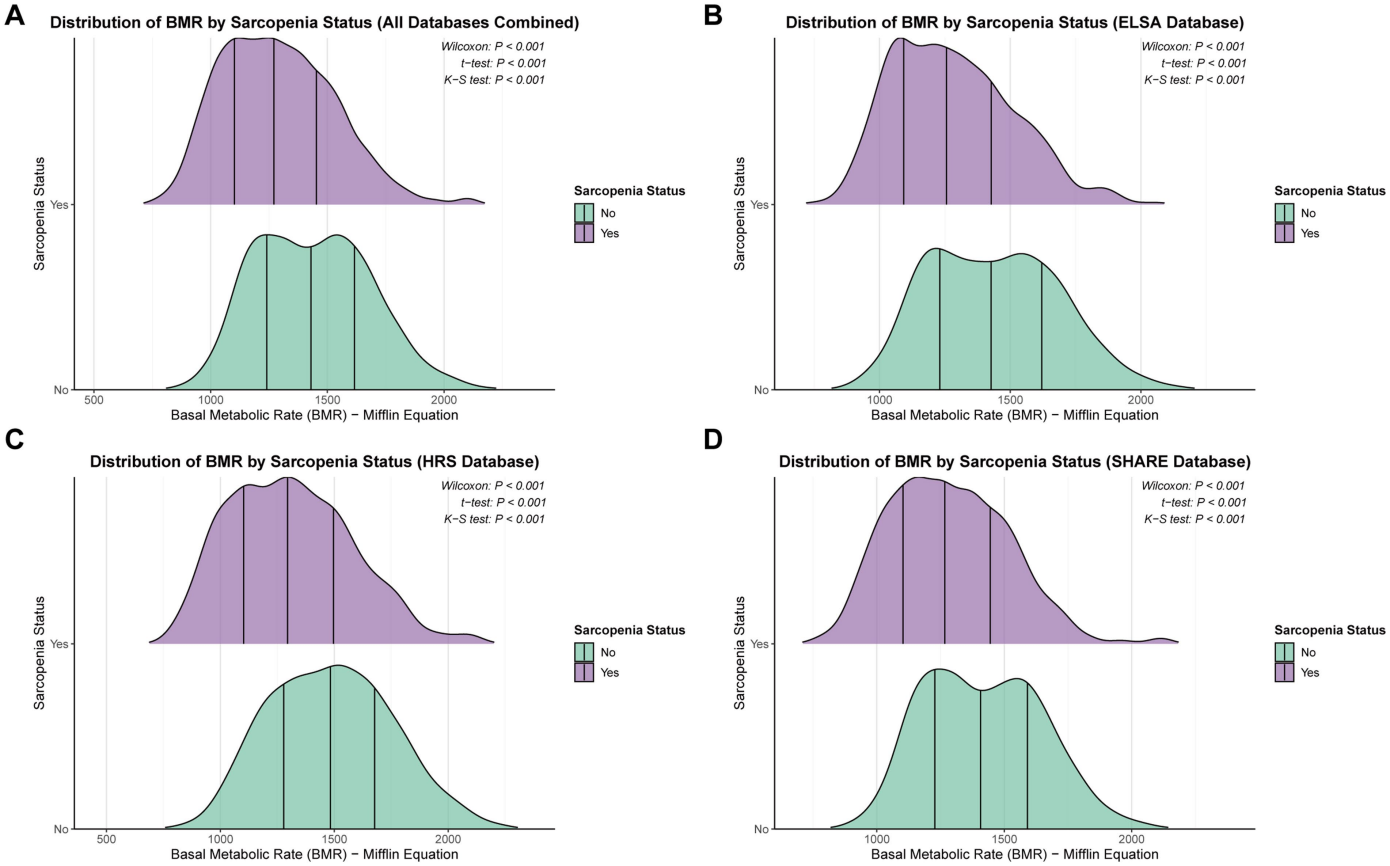
Distribution of basal metabolic rate by sarcopenia status ridge plots showing BMR distribution stratified by sarcopenia status in **(A)** combined cohorts, **(B)** ELSA, **(C)** HRS, and **(D)** SHARE. *p*-values from Wilcoxon rank-sum test, *t*-test, and Kolmogorov–Smirnov test are displayed.

The distribution patterns of BMR differed significantly between participants with and without sarcopenia across all cohorts (Figure 2). In the combined analysis of all databases, participants with sarcopenia demonstrated a leftward shift in BMR distribution compared to those without sarcopenia, with clear separation between the two groups (Figure 2A). This pattern was consistently observed across individual cohorts, with the most pronounced separation in the HRS database (Figure 2C).

Statistical testing confirmed highly significant differences in BMR distributions between sarcopenia groups across all analyses. The Wilcoxon rank-sum test yielded test statistics of 714,955.5 for ELSA, 1,056,776 for HRS, and 5,046,961 for SHARE (all *p* < 0.001). Similarly, independent *t*-tests demonstrated substantial mean differences, with *t*-statistics of 12.038, 12.138, and 15.973 for ELSA, HRS, and SHARE, respectively (all *p* < 0.001). The Kolmogorov–Smirnov test, which examines differences in the entire distribution rather than just central tendency, revealed significant distributional differences with D-statistics of 0.251 (ELSA), 0.253 (HRS), and 0.206 (SHARE), all achieving *p* < 0.001.

The ridge plots visually depicted the magnitude of these differences, showing that participants with sarcopenia had consistently lower BMR values with narrower distributions compared to their non-sarcopenic counterparts. The separation between distributions was most evident in the HRS cohort (Figure 2C), where the sarcopenia group showed minimal overlap with the non-sarcopenia group. These findings underscore the strong inverse relationship between sarcopenia status and basal metabolic rate across diverse aging populations, suggesting that reduced muscle mass is associated with substantial decreases in metabolic capacity.

### Cumulative incidence of osteoporosis by sarcopenia status and BMR quartiles

During a median follow-up of 11.5 years across all cohorts, 1,490 (8.35%) participants developed osteoporosis, with an overall incidence density of 7.72 per 1,000 person-years. The incidence varied substantially across databases, with HRS demonstrating the highest cumulative incidence rate (13.41%), followed by ELSA (10.08%) and SHARE (5.53%). The differences in follow-up duration partially explained these variations, with mean follow-up times of 7.37, 13.26, and 11.56 years for HRS, ELSA, and SHARE, respectively.

**Figure 3:**
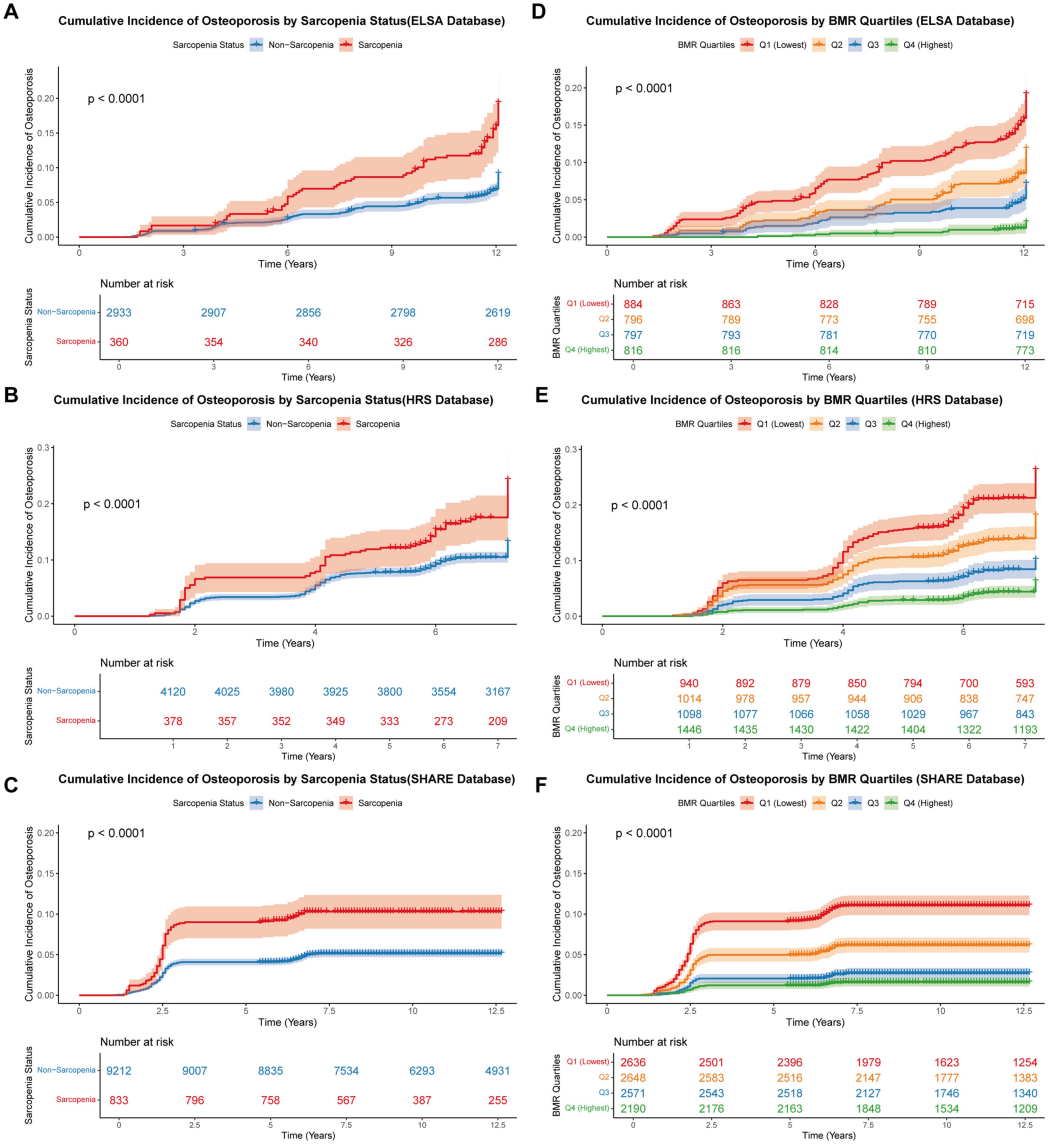
Cumulative incidence curves for osteoporosis. Kaplan–Meier curves showing the cumulative incidence of osteoporosis stratified by sarcopenia status in ELSA **(A)**, HRS **(B)**, and SHARE **(C)**, and by BMR quartiles (Q1–Q4) in ELSA **(D)**, HRS **(E)**, and SHARE **(F)**. *p* values were derived from log-rank tests.

Kaplan–Meier survival analysis revealed significant associations between sarcopenia status and osteoporosis incidence across all three cohorts (Figures 3A–C). Participants with sarcopenia consistently showed higher cumulative incidence of osteoporosis compared to those without sarcopenia. Log-rank tests confirmed these differences were statistically significant, with chi-square values of 37.002 (*p* < 0.0001) for ELSA, 27.669 (*p* < 0.0001) for HRS, and 40.721 (*p* < 0.0001) for SHARE. The separation between curves became evident early in the follow-up period and persisted throughout, with the sarcopenia group maintaining approximately 1.5 to 2-fold higher risk across all databases.

A clear dose–response relationship was observed between BMR quartiles and osteoporosis incidence (Figures 3D–F). Participants in the lowest BMR quartile (Q1) demonstrated the highest cumulative incidence of osteoporosis, while those in the highest quartile (Q4) showed the lowest incidence across all cohorts. This gradient effect was highly significant, with log-rank test chi-square values of 149.349 for ELSA, 215.394 for HRS, and 259.795 for SHARE (all *p* < 0.0001). The inverse association of higher BMR was most pronounced in the SHARE cohort, where the cumulative incidence at 12 years was approximately 20% in Q1 compared to less than 5% in Q4.

The combined analysis of sarcopenia status and BMR quartiles revealed even more pronounced stratification of osteoporosis risk, with chi-square values of 178.012, 226.884, and 285.686 for ELSA, HRS, and SHARE, respectively (all *p* < 0.0001). These findings indicate that both low muscle mass (sarcopenia) and reduced metabolic rate (low BMR) are independent predictors of osteoporosis development, with potential synergistic effects when both conditions coexist.

### Association between BMR quartiles and osteoporosis risk

Prior to the main analysis, multicollinearity assessment was performed to ensure the validity of our regression models (Supplementary Table 1). In Model 1, which included all anthropometric variables, severe multicollinearity was detected with variance inflation factor (VIF) values exceeding 10 for weight (VIF range: 16.3–126.2), BMI (VIF range: 7.2–90.5), BMR (VIF range: 10.8–15.5), and height (VIF range: 5.6–43.5) across all databases. However, in Models 2–4, which included BMR with demographic and clinical covariates, all VIF values were below 2.5, indicating no multicollinearity concerns. The highest VIF in the adjusted models was 2.21 for BMR in SHARE, confirming the appropriateness of including BMR alongside other covariates in our analyses.

**Figure 4:**
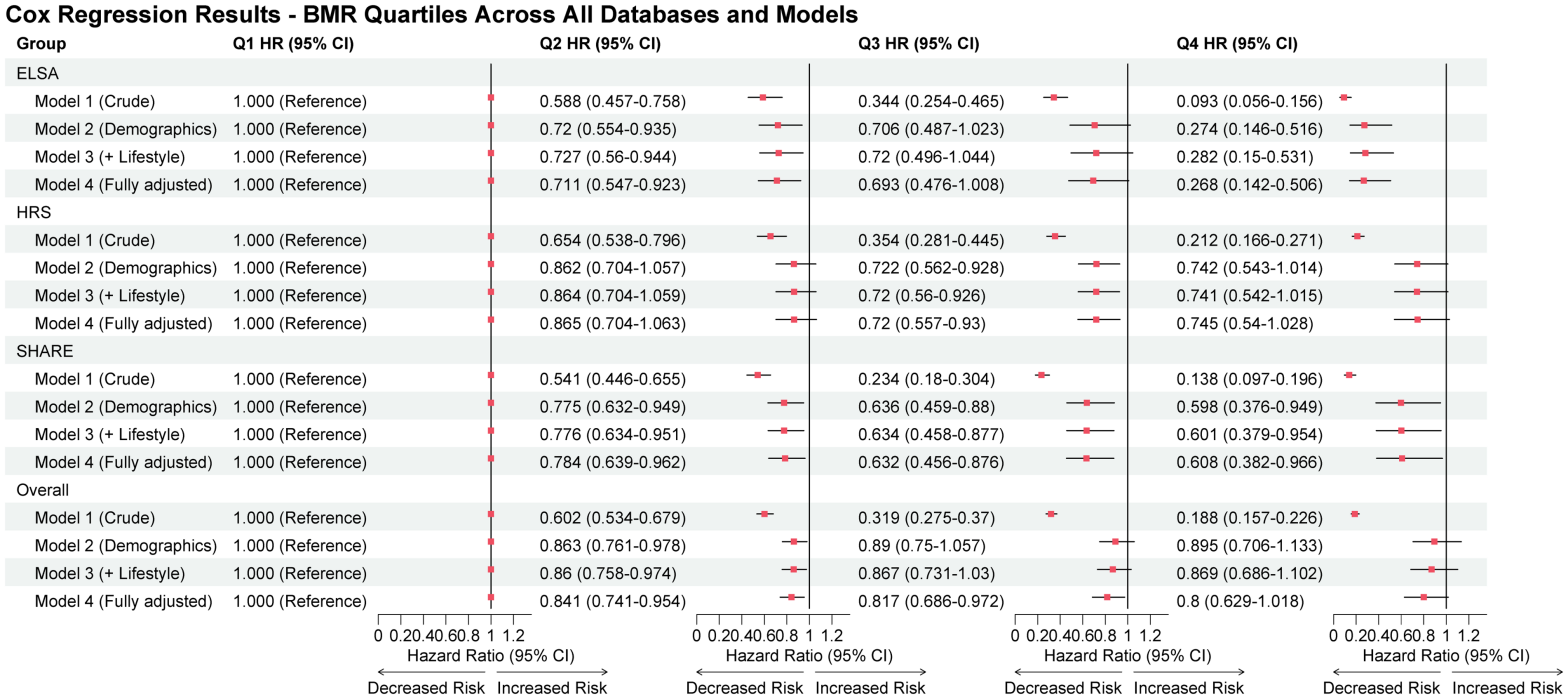
Association between BMR quartiles and osteoporosis risk. Forest plot of hazard ratios (HRs) and 95% confidence intervals (CIs) for incident osteoporosis across BMR quartiles (Q2–Q4 vs. Q1) in the combined sample and in each cohort. Estimates were obtained from Cox proportional hazards models with progressive adjustment (Model 1: crude; Model 2: demographics; Model 3: + lifestyle factors; Model 4: + comorbidities; see Methods for full definitions).

Cox proportional hazards regression revealed a strong inverse association between BMR quartiles and osteoporosis risk (Figure 4). In the crude analysis (Model 1), participants in the highest BMR quartile (Q4) demonstrated markedly reduced osteoporosis risk compared to the lowest quartile (Q1) across all cohorts, with hazard ratios (HRs) of 0.188 (95% CI: 0.157–0.226) in the combined analysis, 0.212 (95% CI: 0.166–0.271) in HRS, 0.093 (95% CI: 0.056–0.156) in ELSA, and 0.138 (95% CI: 0.097–0.196) in SHARE (all *p* < 0.001).

After progressive adjustment for potential confounders, the inverse association of higher BMR persisted, though attenuated. In the fully adjusted model (Model 4), which included demographics, lifestyle factors, and comorbidities, significant inverse association remained evident in the combined analysis (Q2: HR 0.841, 95% CI: 0.741–0.954, *p* = 0.007; Q3: HR 0.817, 95% CI: 0.686–0.972, *p* = 0.023), with a borderline significant trend for Q4 (HR 0.800, 95% CI: 0.629–1.018, *p* = 0.070). The association was most pronounced in ELSA, where Q4 maintained a 73% risk reduction (HR 0.268, 95% CI: 0.142–0.506, *p* < 0.001) even after full adjustment.

Database-specific analyses revealed heterogeneity in the magnitude of associations. ELSA showed the strongest inverse association across all BMR quartiles, followed by SHARE, while HRS demonstrated more modest associations. The model discrimination, as measured by the concordance index, improved from approximately 0.66–0.69 in crude models to 0.72–0.75 in fully adjusted models across all databases, indicating good predictive performance. These findings underscore the independent inverse association of higher basal metabolic rate against osteoporosis development, even after accounting for multiple confounding factors.

### Association pattern between BMR and osteoporosis risk

**Figure 5:**
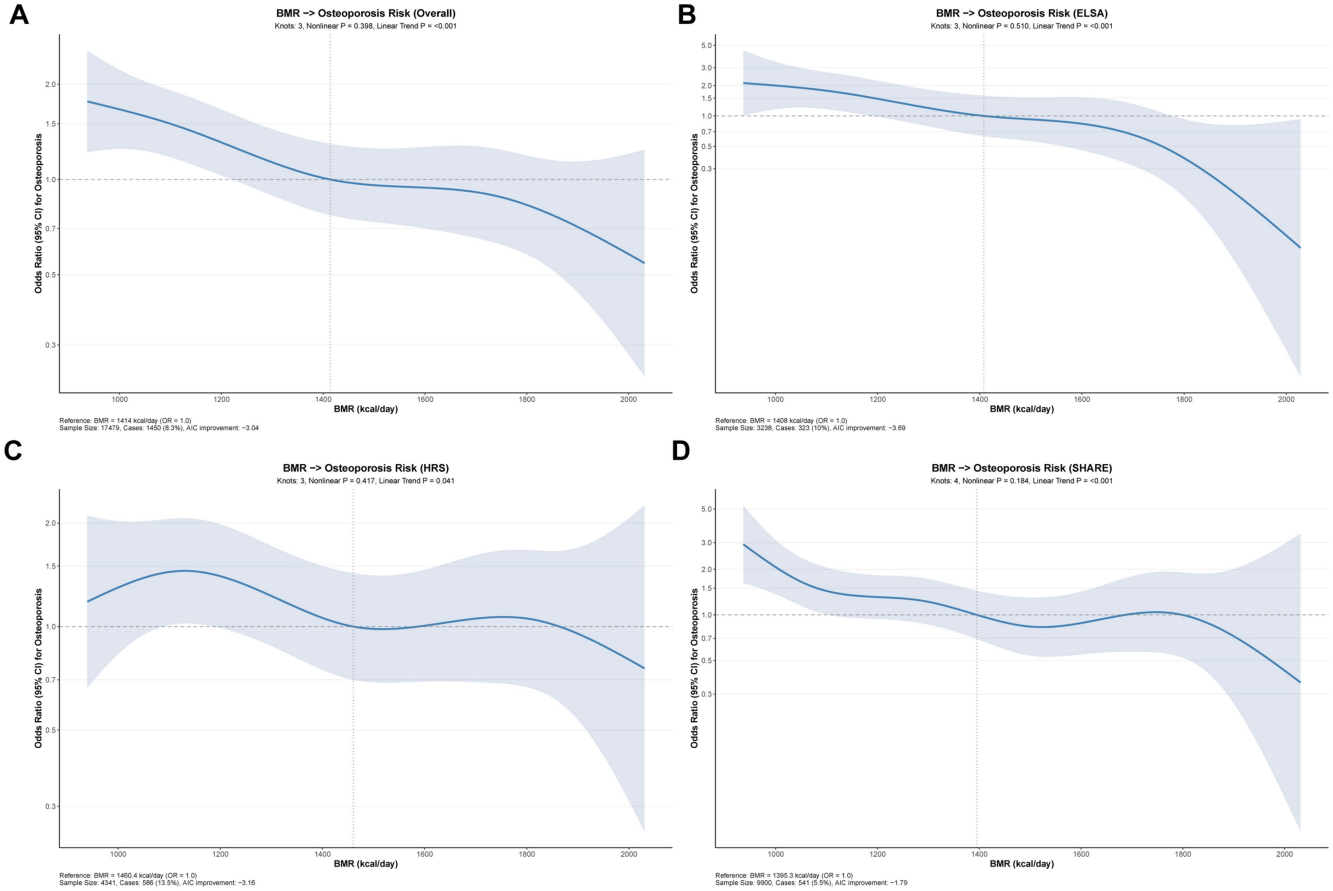
Dose–response relationship between BMR and osteoporosis risk. Restricted cubic spline curves showing the association between continuous BMR and osteoporosis [odds ratio (OR) with 95% CI] in **(A)** combined cohorts, **(B)** ELSA, **(C)** HRS, and **(D)** SHARE. Estimates were adjusted for all covariates (fully adjusted model). The reference was set at the median BMR; shaded areas represent 95% confidence intervals.

Restricted cubic spline analysis was performed to examine the potential non-linear relationship between continuous BMR and osteoporosis risk (Figure 5). The optimal number of knots was determined by the Akaike Information Criterion (AIC), with 3 knots selected for the overall analysis, HRS, and ELSA, while 4 knots were optimal for SHARE. The knot positions varied across databases, reflecting the heterogeneous BMR distributions: overall (1120.1, 1,414, 1743.2 kcal/day), HRS (1128.8, 1460.4, 1804.6 kcal/day), ELSA (1,114, 1,408, 1741.4 kcal/day), and SHARE (1066.5, 1287.5, 1,510, 1788.7 kcal/day).

Despite the flexibility of the spline models, likelihood ratio tests revealed no significant departure from linearity in any of the analyses. The non-linearity *p*-values were 0.398 for the overall analysis, 0.417 for HRS, 0.510 for ELSA, and 0.184 for SHARE, all exceeding the significance threshold. The AIC improvements from adding spline terms were minimal, ranging from −1.79 to −3.69, further supporting the absence of meaningful non-linear associations. These findings indicate that the relationship between BMR and osteoporosis risk is predominantly linear across the observed BMR range (approximately 936–2031 kcal/day).

The linear trend analysis confirmed a significant inverse association between BMR and osteoporosis risk. In the overall analysis (Figure 5A), each unit increase in BMR was associated with a 0.1% reduction in osteoporosis odds (OR = 0.999, *p* < 0.001). This protective linear trend was consistent across individual databases: ELSA demonstrated the strongest effect (OR = 0.9982, *p* < 0.001; Figure 5B), followed by SHARE (OR = 0.9988, *p* < 0.001; Figure 5D), while HRS showed a slightly weaker but still significant association (OR = 0.9994, *p* = 0.041; Figure 5C).

The visual inspection of the spline curves revealed a generally monotonic decrease in osteoporosis risk with increasing BMR across all databases. The confidence intervals remained relatively narrow throughout most of the BMR range, widening only at the extremes where data were sparse. Notably, the inverse association appeared most pronounced in ELSA, where the curve showed a steeper decline, particularly at higher BMR values. These findings support the use of linear models in examining the BMR-osteoporosis association and indicate that higher BMR is associated with lower osteoporosis risk across the entire metabolic rate spectrum.

### Subgroup analyses and effect modification of BMR-osteoporosis association

**Figure 6:**
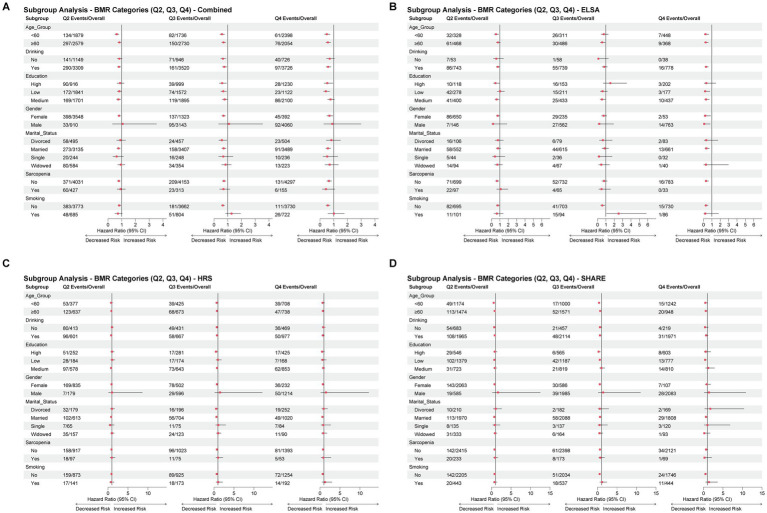
Subgroup analyses of the association between BMR quartiles and osteoporosis. Forest plots displaying hazard ratios (HRs) and 95% confidence intervals for BMR quartiles (Q2–Q4 vs. Q1) across subgroups in **(A)** combined cohorts, **(B)** ELSA, **(C)** HRS, and **(D)** SHARE. *p* values for interaction are shown on the right.

Comprehensive subgroup analyses were conducted to identify inverse association modifiers of the BMR-osteoporosis relationship across various demographic and clinical characteristics (Figure 6). In the combined cohort analysis, the inverse association of higher BMR (per 1 SD increase) on osteoporosis risk was consistently observed across most subgroups, with hazard ratios ranging from 0.723 to 0.924. Notably, the protective association was more pronounced in younger participants (<60 years: HR 0.723, 95% CI: 0.626–0.836) compared to older participants (≥60 years: HR 0.791, 95% CI: 0.721–0.869), although the interaction was not statistically significant (P-interaction = 0.565).

Significant effect modification was identified for education level and smoking status in the combined analysis. The interaction with education (P-interaction = 0.007) revealed that the inverse association of BMR was strongest among participants with low education (HR 0.758, 95% CI: 0.657–0.875), intermediate among those with medium education (HR 0.890, 95% CI: 0.792–1.002), and weakest but still significant among highly educated participants (HR 0.747, 95% CI: 0.613–0.908). For smoking status, a significant interaction was observed (P-interaction = 0.011), with non-smokers showing a stronger inverse association (HR 0.793, 95% CI: 0.725–0.867) compared to current smokers (HR 1.001, 95% CI: 0.819–1.225), suggesting that smoking may attenuate the association of higher BMR.

Database-specific analyses revealed heterogeneous interaction patterns. In HRS, education showed significant effect modification (P-interaction = 0.041), with the inverse association being most evident among participants with high education (HR 0.795, 95% CI: 0.617–1.023). In ELSA, drinking status emerged as a significant effect modifier (P-interaction = 0.032), where the inverse association was substantially stronger among non-drinkers (HR 0.357, 95% CI: 0.156–0.815) compared to drinkers (HR 0.686, 95% CI: 0.562–0.838). SHARE showed no significant interactions, although trends suggested potential effect modification by age (P-interaction = 0.055) and smoking (P-interaction = 0.086).

Across all databases, the BMR-osteoporosis association remained relatively consistent across gender, marital status, and sarcopenia status, with no significant interactions detected. The inverse association was maintained in both sarcopenic and non-sarcopenic participants, suggesting that the association of higher BMR on bone health operate independently of muscle mass status. These findings indicate that while the overall inverse association of BMR on osteoporosis risk is robust across diverse populations, certain lifestyle factors, particularly smoking and drinking, may modulate this relationship, highlighting the importance of comprehensive risk assessment in osteoporosis prevention strategies.

### Sensitivity analyses for BMR-osteoporosis association

Multiple sensitivity analyses were performed to evaluate the robustness of the observed associations between BMR and osteoporosis risk (Supplementary Tables 2, 3). The inverse association of higher BMR remained consistent across all sensitivity analyses, strengthening confidence in the primary findings.

When excluding early events occurring within 24 months of baseline to minimize potential reverse causation, the dose–response relationship persisted with BMR quartiles showing progressively lower hazard ratios: Q2 (HR 0.807, 95% CI: 0.702–0.927, *p* = 0.003), Q3 (HR 0.736, 95% CI: 0.608–0.890, *p* = 0.002), and Q4 (HR 0.652, 95% CI: 0.502–0.846, *p* = 0.001) compared to Q1. Similarly, the continuous BMR analysis demonstrated a robust inverse association (HR 0.831, 95% CI: 0.759–0.909, *p* < 0.001 per 1 SD increase) among 17,582 participants with 1,236 events after excluding early outcomes.

The exclusion of extreme BMR values (beyond 1st and 99th percentiles) to address potential measurement errors or outliers yielded comparable results. The quartile analysis showed significant inverse associations across all higher quartiles (Q2: HR 0.791, *p* < 0.001; Q3: HR 0.709, *p* < 0.001; Q4: HR 0.641, *p* < 0.001), while continuous BMR analysis confirmed the association (HR 0.808, 95% CI: 0.744–0.877, *p* < 0.001). Complete case analysis, utilizing only participants with no missing data (n = 17,836, events = 1,490), demonstrated even stronger associations, with the highest quartile showing a 37% risk reduction (HR 0.629, 95% CI: 0.494–0.800, *p* < 0.001).

Database-specific analyses revealed consistent inverse associations across all three cohorts, though with varying magnitudes. ELSA showed the strongest association (HR 0.648, 95% CI: 0.534–0.785, *p* < 0.001), followed by SHARE (HR 0.777, 95% CI: 0.666–0.906, *p* = 0.001) and HRS (HR 0.890, 95% CI: 0.793–0.998, *p* = 0.046). Time-stratified analyses at different follow-up cutpoints demonstrated the persistence of the inverse association over time, with significant associations observed at both 36 months (HR 0.807, 95% CI: 0.709–0.920, *p* = 0.001) and 60 months (HR 0.800, 95% CI: 0.720–0.890, *p* < 0.001) of follow-up.

Gender-stratified analyses revealed that the inverse association was significant in females (HR 0.811, 95% CI: 0.742–0.886, *p* < 0.001) but not in males (HR 0.916, 95% CI: 0.744–1.129, *p* = 0.411), although the limited number of events in males (*n* = 223) may have reduced statistical power. Overall, these comprehensive sensitivity analyses confirm the robustness of the inverse association between BMR and osteoporosis risk, with the inverse association remaining significant across various analytical approaches and population subsets.

## Discussion

Based on three major international aging cohorts, our study consistently demonstrated an inverse association between higher basal metabolic rate (BMR) and lower risk of osteoporosis. This relationship remained robust across a wide range of statistical models, alternative exposure definitions, methods for handling missing data, and sensitivity analyses. The consistent direction and magnitude of association across all three datasets and subgroups stratified by sex and age further support the stability of the findings. Notably, we observed greater marginal benefits among individuals with lower educational attainment and current smokers, suggesting that socioeconomic and behavioral exposures—such as reduced energy availability, lower dietary quality, and limited health literacy—may exacerbate skeletal vulnerability. These findings suggest that BMR may serve as a useful marker for identifying individuals at higher risk in these subpopulations. From a clinical perspective, BMR represents an accessible and quantifiable metabolic phenotype that may complement existing risk assessment tools in early identification of high-risk individuals. However, whether interventions targeting BMR can causally reduce osteoporosis risk remains to be established through randomized controlled trials.

Mechanistically, BMR reflects a composite phenotype of systemic energy supply–demand balance and tissue metabolic activity, and has been hypothesized to be linked with bone remodeling through multiple interrelated pathways. Biologically, several pathways may potentially link BMR with bone health, though these remain speculative. Higher BMR is associated with enhanced mitochondrial function and AMPK–mTOR–SIRT1/PGC-1α signaling, which have been implicated in osteoblast activity and bone formation (5, 12, 13). BMR is also correlated with fat-free mass and muscle quality; mechanical loading from muscle contractions and myokine secretion (e.g., IGF-1, irisin) have been linked to bone metabolism through mechanoresponsive and endocrine pathways (14–16).

Additionally, higher BMR has been associated with improved insulin sensitivity, reduced systemic inflammation, and favorable adipokine profiles, all of which may influence the bone marrow microenvironment and osteoblast–osteoclast balance (17–19). Endocrine factors including thyroid hormones and the growth hormone/IGF-1 axis, which regulate both metabolic rate and bone turnover, may also contribute to this relationship (20–23). However, these mechanisms are derived largely from experimental studies and cross-sectional observations (24–27), and whether they mediate the BMR-osteoporosis association observed in our study cannot be determined from our data (28–30).

From a clinical and public health standpoint, BMR represents an accessible metabolic phenotype that may complement traditional tools such as DXA and FRAX in risk stratification. Our findings indicate that individuals with lower BMR are at higher risk of osteoporosis, and that this association is particularly pronounced in certain subgroups (lower education, current smokers). However, it is critical to note that our observational design cannot determine whether interventions aimed at increasing BMR would causally reduce osteoporosis risk. While resistance training and nutritional interventions can increase BMR and have been associated with bone health benefits in some studies (31). Randomized controlled trials specifically testing whether BMR-enhancing interventions reduce fracture incidence are needed before clinical recommendations can be made (32–35).

Future studies should employ instrumental variable approaches and mediation analyses to strengthen causal inference and use indirect calorimetry or wearable devices for accurate metabolic phenotyping. Randomized controlled trials focusing on BMR enhancement should assess impacts on bone mass, fracture incidence, and functional outcomes. Additionally, integrating BMR into extended osteoporosis risk prediction models may improve discriminatory accuracy and clinical utility. In summary, the robust and biologically plausible association between higher BMR and lower osteoporosis risk supports a metabolism-oriented prevention framework and provides clear mechanistic targets for future intervention.

### Strengths and limitations

This study presents several methodological strengths. It is one of the first to evaluate the longitudinal association between BMR and osteoporosis risk using three large, independent, and geographically diverse aging cohorts. This design enhances the external validity and generalizability of our findings. The exposure was modeled both as a categorical (quartile-based) and continuous variable, with restricted cubic splines applied to capture potential nonlinear dose–response relationships and thresholds. We also undertook comprehensive confounding control, including adjustment for demographics, comorbidities, lifestyle factors, and sarcopenia-related variables. Robustness was further ensured through sensitivity analyses that excluded early events and extreme values, used complete-case datasets, and performed separate validations within each cohort.

Nonetheless, several limitations should be acknowledged:

1) Outcome ascertainment: incident osteoporosis was based on self-reported physician diagnosis rather than objective DXA/fracture records, which may introduce misclassification.
2) Exposure measurement: BMR was estimated using the Mifflin–St Jeor equation instead of direct calorimetry, which may cause measurement error and attenuation of associations.
3) Cohort heterogeneity: sarcopenia definitions, questionnaires, and follow-up schedules differed across ELSA, HRS, and SHARE, and residual inconsistencies may remain despite harmonization.
4) Residual confounding: unmeasured or imperfectly measured factors (e.g., diet, vitamin D/calcium supplementation, medication use, biomarkers, physical activity intensity, and genetics) could bias effect estimates.
5) Observational nature: reverse causation and time-varying confounding cannot be fully excluded; therefore, causal inferences should be avoided.

Despite these limitations, several features of our study strengthen confidence in the robustness of the observed association. Most importantly, the inclusion of three large, independent, and geographically diverse cohorts (European, North American) provides cross-validation of the BMR-osteoporosis association. The consistency of findings across these populations—despite differences in healthcare systems, dietary patterns, measurement protocols, and follow-up procedures—argues against the association being an artifact of any single cohort’s specific methodology or population characteristics. Additionally, the dose–response pattern observed across BMR quartiles, the persistence of associations after comprehensive covariate adjustment, and the stability of results across multiple sensitivity analyses all support the validity of our findings. While causality cannot be established from observational data alone, the convergence of evidence across multiple independent populations provides stronger support for a genuine association than would be possible from a single cohort study.

## Conclusion

Across three large, well-characterized aging cohorts (ELSA, HRS, SHARE), higher basal metabolic rate (BMR) was independently associated with a lower risk of incident osteoporosis. This association was approximately linear across the observed BMR range and remained consistent after extensive covariate adjustment, multiple sensitivity analyses, and cohort-specific validations. The strength of association varied by education and smoking status, suggesting potential effect modification by socioeconomic and behavioral factors.

Importantly, as an observational study, our findings cannot establish causality. The observed association may reflect the direct metabolic influence of BMR on bone health, but could also be explained by residual confounding, measurement error, or reverse causation. The consistency of findings across three independent international cohorts strengthens confidence in a genuine association but does not eliminate these alternative explanations.

From a clinical perspective, BMR represents an accessible metabolic phenotype that may complement traditional assessment tools (e.g., DXA, FRAX) in identifying individuals at higher osteoporosis risk, particularly in vulnerable subgroups. However, whether interventions that increase or preserve BMR—such as resistance training, optimized nutrition, or metabolic health programs—can causally reduce osteoporosis incidence remains unknown and requires testing in randomized controlled trials.

Future research priorities include: (1) validating these associations using directly measured energy expenditure (e.g., indirect calorimetry, doubly labeled water) rather than equation-derived estimates; (2) employing objective skeletal outcomes (DXA-measured bone mineral density, radiographically confirmed fractures) rather than self-reported diagnoses; (3) conducting mechanistic studies to identify biological pathways linking BMR to bone metabolism; and (4) implementing randomized controlled trials of BMR-enhancing interventions with fracture outcomes to establish causality. Only through such rigorous investigation can we determine whether BMR is a modifiable causal factor or simply a marker of osteoporosis risk.

## Data Availability

The original contributions presented in the study are included in the article/Supplementary material, further inquiries can be directed to the corresponding authors.

## Glossary

AMPK: AMP-activated protein kinase
BMR: basal metabolic rate
BMI: body mass index
CI: confidence interval
DXA: dual-energy X-ray absorptiometry
ELSA: English Longitudinal Study of Ageing
FGF21: fibroblast growth factor 21
FRAX: Fracture Risk Assessment Tool
GH: growth hormone
HR: hazard ratio
HRS: Health and Retirement Study
IGF-1: insulin-like growth factor 1
IL-6: interleukin-6
IQR: interquartile range
mTOR: mechanistic target of rapamycin
OPG: osteoprotegerin
PGC-1*α*: peroxisome proliferator-activated receptor-*γ* coactivator-1α
Piezo1: Piezo-type mechanosensitive ion channel component 1
PPARγ: peroxisome proliferator-activated receptor-γ
RANKL: receptor activator of nuclear factor-κB ligand
RCS: restricted cubic spline
ROS: reactive oxygen species
SD: standard deviation
SHARE: Survey of Health, Ageing and Retirement in Europe
SIRT1: sirtuin 1
SMD: standardized mean difference
TNF-α: tumor necrosis factor-α
VIF: variance inflation factor
YAP/TAZ: Yes-associated protein/transcriptional coactivator with PDZ-binding motif
