# Supplementary material for: Basal metabolic rate as a protective factor against osteoporosis: a multi-cohort longitudinal study from three international aging databases
Source: Front Nutr. 2026 Jan 22;13:1712489. doi: 10.3389/fnut.2026.1712489 (PMC12872545; doi:10.3389/fnut.2026.1712489)
Supplement: Supplementary file 1 [file Table_1.docx]

**Supplementary Table 1. Multicollinearity assessment using variance inflation factors**

| Database | Model | Variable | VIF | Tolerance | Multicollinearity Level | Action Needed |
| --- | --- | --- | --- | --- | --- | --- |
| Overall | model1 | Weight Kg | 24.503 | 0.0408 | Severe (>10) | Remove variable |
| Overall | model1 | BMI | 15 | 0.0667 | Severe (>10) | Remove variable |
| Overall | model1 | BMR Mifflin | 12.366 | 0.0809 | Severe (>10) | Remove variable |
| Overall | model1 | Height Meters | 8.914 | 0.1122 | High (5-10) | Consider removal |
| Overall | model2 | BMR Mifflin | 2.01 | 0.4974 | Low (<2.5) | Accept |
| Overall | model2 | Gender num | 1.98 | 0.5051 | Low (<2.5) | Accept |
| Overall | model2 | Education num | 1.025 | 0.9761 | Low (<2.5) | Accept |
| Overall | model3 | BMR Mifflin | 2.01 | 0.4974 | Low (<2.5) | Accept |
| Overall | model3 | Gender num | 2.004 | 0.4991 | Low (<2.5) | Accept |
| Overall | model3 | Education num | 1.048 | 0.9542 | Low (<2.5) | Accept |
| Overall | model3 | Drinking num | 1.048 | 0.9541 | Low (<2.5) | Accept |
| Overall | model3 | Smoking num | 1.007 | 0.9927 | Low (<2.5) | Accept |
| Overall | model4 | BMR Mifflin | 2.01 | 0.4974 | Low (<2.5) | Accept |
| Overall | model4 | Gender num | 2.004 | 0.4991 | Low (<2.5) | Accept |
| Overall | model4 | Education num | 1.048 | 0.9542 | Low (<2.5) | Accept |
| Overall | model4 | Drinking num | 1.048 | 0.9541 | Low (<2.5) | Accept |
| Overall | model4 | Smoking num | 1.007 | 0.9927 | Low (<2.5) | Accept |
| HRS | model1 | Weight Kg | 16.338 | 0.0612 | Severe (>10) | Remove variable |
| HRS | model1 | BMR Mifflin | 13.855 | 0.0722 | Severe (>10) | Remove variable |
| HRS | model1 | BMI | 7.178 | 0.1393 | High (5-10) | Consider removal |
| HRS | model1 | Height Meters | 5.596 | 0.1787 | High (5-10) | Consider removal |
| HRS | model2 | BMR Mifflin | 1.742 | 0.574 | Low (<2.5) | Accept |
| HRS | model2 | Gender num | 1.728 | 0.5788 | Low (<2.5) | Accept |
| HRS | model2 | Education num | 1.013 | 0.987 | Low (<2.5) | Accept |
| HRS | model3 | Gender num | 1.745 | 0.5731 | Low (<2.5) | Accept |
| HRS | model3 | BMR Mifflin | 1.743 | 0.5738 | Low (<2.5) | Accept |
| HRS | model3 | Education num | 1.061 | 0.9423 | Low (<2.5) | Accept |
| HRS | model3 | Drinking num | 1.056 | 0.9471 | Low (<2.5) | Accept |
| HRS | model3 | Smoking num | 1.019 | 0.9813 | Low (<2.5) | Accept |
| HRS | model4 | Gender num | 1.745 | 0.5731 | Low (<2.5) | Accept |
| HRS | model4 | BMR Mifflin | 1.743 | 0.5738 | Low (<2.5) | Accept |
| HRS | model4 | Education num | 1.061 | 0.9423 | Low (<2.5) | Accept |
| HRS | model4 | Drinking num | 1.056 | 0.9471 | Low (<2.5) | Accept |
| HRS | model4 | Smoking num | 1.019 | 0.9813 | Low (<2.5) | Accept |
| ELSA | model1 | Weight Kg | 126.151 | 0.0079 | Severe (>10) | Remove variable |
| ELSA | model1 | BMI | 90.485 | 0.0111 | Severe (>10) | Remove variable |
| ELSA | model1 | Height Meters | 43.226 | 0.0231 | Severe (>10) | Remove variable |
| ELSA | model1 | BMR Mifflin | 15.462 | 0.0647 | Severe (>10) | Remove variable |
| ELSA | model2 | Gender num | 2.164 | 0.4622 | Low (<2.5) | Accept |
| ELSA | model2 | BMR Mifflin | 2.164 | 0.4621 | Low (<2.5) | Accept |
| ELSA | model2 | Education num | 1.034 | 0.9668 | Low (<2.5) | Accept |
| ELSA | model3 | Gender num | 2.166 | 0.4618 | Low (<2.5) | Accept |
| ELSA | model3 | BMR Mifflin | 2.165 | 0.4619 | Low (<2.5) | Accept |
| ELSA | model3 | Education num | 1.052 | 0.9505 | Low (<2.5) | Accept |
| ELSA | model3 | Drinking num | 1.011 | 0.9887 | Low (<2.5) | Accept |
| ELSA | model3 | Smoking num | 1.01 | 0.9897 | Low (<2.5) | Accept |
| ELSA | model4 | Gender num | 2.166 | 0.4618 | Low (<2.5) | Accept |
| ELSA | model4 | BMR Mifflin | 2.165 | 0.4619 | Low (<2.5) | Accept |
| ELSA | model4 | Education num | 1.052 | 0.9505 | Low (<2.5) | Accept |
| ELSA | model4 | Drinking num | 1.011 | 0.9887 | Low (<2.5) | Accept |
| ELSA | model4 | Smoking num | 1.01 | 0.9897 | Low (<2.5) | Accept |
| SHARE | model1 | Weight Kg | 124.345 | 0.008 | Severe (>10) | Remove variable |
| SHARE | model1 | BMI | 85.303 | 0.0117 | Severe (>10) | Remove variable |
| SHARE | model1 | Height Meters | 43.547 | 0.023 | Severe (>10) | Remove variable |
| SHARE | model1 | BMR Mifflin | 10.83 | 0.0923 | Severe (>10) | Remove variable |
| SHARE | model2 | BMR Mifflin | 2.201 | 0.4544 | Low (<2.5) | Accept |
| SHARE | model2 | Gender num | 2.175 | 0.4597 | Low (<2.5) | Accept |
| SHARE | model2 | Education num | 1.02 | 0.9807 | Low (<2.5) | Accept |
| SHARE | model3 | BMR Mifflin | 2.209 | 0.4526 | Low (<2.5) | Accept |
| SHARE | model3 | Gender num | 2.193 | 0.456 | Low (<2.5) | Accept |
| SHARE | model3 | Drinking num | 1.095 | 0.9129 | Low (<2.5) | Accept |
| SHARE | model3 | Education num | 1.064 | 0.9402 | Low (<2.5) | Accept |
| SHARE | model3 | Smoking num | 1.01 | 0.9905 | Low (<2.5) | Accept |
| SHARE | model4 | BMR Mifflin | 2.209 | 0.4526 | Low (<2.5) | Accept |
| SHARE | model4 | Gender num | 2.193 | 0.456 | Low (<2.5) | Accept |
| SHARE | model4 | Drinking num | 1.095 | 0.9129 | Low (<2.5) | Accept |
| SHARE | model4 | Education num | 1.064 | 0.9402 | Low (<2.5) | Accept |
| SHARE | model4 | Smoking num | 1.01 | 0.9905 | Low (<2.5) | Accept |
