# Supplementary material for: Basal metabolic rate as a protective factor against osteoporosis: a multi-cohort longitudinal study from three international aging databases
Source: Front Nutr. 2026 Jan 22;13:1712489. doi: 10.3389/fnut.2026.1712489 (PMC12872545; doi:10.3389/fnut.2026.1712489)
Supplement: Supplementary file 2 [file Table_2.docx]

**Supplementary Table 2. Sensitivity analysis for BMR quartiles and osteoporosis risk**

| Analysis Type | BMR Category | HR CI | P formatted | N | Events |
| --- | --- | --- | --- | --- | --- |
| Exclude Early Events | Q1 | 1.000 (Reference) | Reference | 17582 | 1236 |
| Exclude Early Events | Q2 | 0.807 (0.702-0.927) | 0.003 | 17582 | 1236 |
| Exclude Early Events | Q3 | 0.736 (0.608-0.89) | 0.002 | 17582 | 1236 |
| Exclude Early Events | Q4 | 0.652 (0.502-0.846) | 0.001 | 17582 | 1236 |
| Exclude Extreme BMR | Q1 | 1.000 (Reference) | Reference | 17479 | 1450 |
| Exclude Extreme BMR | Q2 | 0.791 (0.696-0.899) | <0.001 | 17479 | 1450 |
| Exclude Extreme BMR | Q3 | 0.709 (0.595-0.845) | <0.001 | 17479 | 1450 |
| Exclude Extreme BMR | Q4 | 0.641 (0.505-0.813) | <0.001 | 17479 | 1450 |
| Complete Cases Only | Q1 | 1.000 (Reference) | Reference | 17836 | 1490 |
| Complete Cases Only | Q2 | 0.81 (0.714-0.919) | 0.001 | 17836 | 1490 |
| Complete Cases Only | Q3 | 0.723 (0.607-0.861) | <0.001 | 17836 | 1490 |
| Complete Cases Only | Q4 | 0.629 (0.494-0.8) | <0.001 | 17836 | 1490 |
