# Supplementary material for: Basal metabolic rate as a protective factor against osteoporosis: a multi-cohort longitudinal study from three international aging databases
Source: Front Nutr. 2026 Jan 22;13:1712489. doi: 10.3389/fnut.2026.1712489 (PMC12872545; doi:10.3389/fnut.2026.1712489)
Supplement: Supplementary file 3 [file Table_3.docx]

**Supplementary Table 3. Sensitivity analysis for continuous BMR and osteoporosis risk**

| Analysis Type | BMR Category | HR CI | P | N | Events | Follow up months |
| --- | --- | --- | --- | --- | --- | --- |
| Exclude Early Events | Per 1 SD increase | 0.831 (0.759-0.909) | <0.001 | 17582 | 1236 |  |
| Exclude Extreme BMR | Per 1 SD increase | 0.808 (0.744-0.877) | <0.001 | 17479 | 1450 |  |
| Complete Cases Only | Per 1 SD increase | 0.823 (0.758-0.893) | <0.001 | 17836 | 1490 |  |
| Database HRS | Per 1 SD increase | 0.89 (0.793-0.998) | 0.046 | 4498 | 603 |  |
| Database ELSA | Per 1 SD increase | 0.648 (0.534-0.785) | <0.001 | 3293 | 332 |  |
| Database SHARE | Per 1 SD increase | 0.777 (0.666-0.906) | 0.001 | 10045 | 555 |  |
| Follow up 36months | Per 1 SD increase | 0.807 (0.709-0.92) | 0.001 | 17836 | 646 | 36 |
| Follow up 60months | Per 1 SD increase | 0.8 (0.72-0.89) | <0.001 | 17836 | 892 | 60 |
| Gender Female | Per 1 SD increase | 0.811 (0.742-0.886) | <0.001 | 9662 | 1267 |  |
| Gender Male | Per 1 SD increase | 0.916 (0.744-1.129) | 0.411 | 8174 | 223 |  |
